# Sense of coherence as a pathway linking war trauma and post-migration stress to mental health functioning among refugees and asylum seekers in the Netherlands

**DOI:** 10.3389/fpsyt.2026.1890660

**Published:** 2026-07-15

**Authors:** Elizabeth Nolan, Zoe Vos Coronato, Antoine van Sint Fiet, Tim Wind, Simone de la Rie

**Affiliations:** 1 Department of Clinical Psychology, Faculty of Psychology, Open Universiteit, Heerlen, Netherlands; 2 ARQ Centrum’45, Diemen, Netherlands; 3 ARQ National Psychotrauma Centrum, Diemen, Netherlands

**Keywords:** asylum seekers, mediation, mental health functioning, post-migration stress, refugees, sense of coherence, trauma

## Abstract

**Introduction:**

Refugees carry a cumulative burden of past trauma and ongoing post-migration stress, yet the mechanisms linking these to mental health functioning remain incompletely understood. Sense of coherence — a salutogenic orientation comprising comprehensibility, manageability, and meaningfulness — may help account for these associations. We tested sense of coherence as a mediator of both associations and compared the relative weight of the two stressor domains.

**Methods:**

A cross-sectional sample of 151 refugees and asylum seekers in the Netherlands (84.1% with a residence permit) completed the LEC-5, PMLP, SOC-13, and OQ-45.2 in person with research-assistant support across Dutch, English, and Arabic. We conducted multiple regression and three PROCESS Model 4 mediation analyses, including a parallel mediation with sense of coherence and post-migration stress as simultaneous mediators.

**Results:**

Both stressors predicted poorer functioning, with post-migration stress substantially stronger than trauma exposure (b = .40 vs. .17). Sense of coherence was the strongest correlate of functioning (r = –.57) and statistically mediated both cross-sectional associations. In the parallel mediation, sense of coherence and post-migration stress mediated the trauma–functioning association, and the direct effect was no longer statistically significant once both were included. Family-oriented stressors dominated the burden profile: worries about family in the country of origin (78.8%), missing family (73.5%), and uncertainty about the future (70.9%).

**Discussion:**

As a personal-resilience construct shaped by — not insulated from — external conditions, sense of coherence warrants sustained attention in this population.

## 1 Introduction

More people are forcibly displaced today than at any point in recent history: at mid-2025, an estimated 117.3 million people had fled their homes worldwide, including 42.5 million refugees (44). Refugees often carry a complex burden of past and ongoing adversity. Meta-analyses consistently place the prevalence of PTSD, depression, and anxiety in adult refugees substantially above that of host populations, with pooled estimates around 30% for both PTSD and depression (1–3). Comorbidity among these conditions is common rather than exceptional (4, 5). Yet despite these elevated risks, most refugees do not develop chronic psychopathology and many demonstrate resilience over time (6, 7).

Refugees have been exposed to potentially traumatic events (PTEs) in the country of origin, during forced migration, and continue to face persistent adversities during resettlement (8). Pre-migration exposures typically include armed combat, persecution, imprisonment, torture, and witnessing violence or death. The migration itself can carry further violence — physical assault, deception by smugglers, human trafficking, and sexual violence — alongside the disruption of leaving established social networks, societal roles, and family ties for the unknown.

In the host country, an unfamiliar environment imposes multiple chronic stressors spanning asylum-related (prolonged procedures, legal uncertainty), socioeconomic (unemployment, inadequate housing, barriers to education), social (isolation, discrimination, language difficulties), and adaptation domains (loss of status and professional identity) (9–11). Substantial evidence indicates that such post-migration stressors are often more strongly associated with psychological distress than past traumatic events (12, 13). Unlike discrete past events, these stressors persist, continuously taxing adaptive resources and interfering with recovery (14). They also do not operate independently of past trauma. Prior exposure can heighten vulnerability to ongoing adversity, and fear for family who remain in the country of origin extends pre-migration concerns into the post-migration period (10).

Conservation of Resources theory (15, 38) provides the theoretical lens for why ongoing post-migration adversity weighs so heavily on refugee functioning. The theory holds that psychological well-being depends on the availability and maintenance of resources — material, social, and personal — that people draw on to meet life’s demands, and that adaptive capacity erodes when these resources are chronically depleted faster than they can be replenished. Post-migration conditions for refugees represent exactly such a sustained loss process. Uncertain legal status, underemployment relative to prior qualifications, isolation from networks of origin, and prolonged separation from family together amount to a loss of social capital. This produces continuous resource drain rather than discrete shocks. This loss-spiral logic helps explain why post-migration stressors are typically more strongly associated with psychological distress than past traumatic events (12, 13): they continue to deplete the adaptive resources needed for recovery (14).

Refugees’ capacity to withstand post-migration adversity is shaped not only by the resources available to them but also by their orientation toward what they experience. Sense of coherence (16) captures this individual dimension and functions as a personal-resilience construct. Antonovsky (16, 17) formalized it as a global personal orientation comprising *comprehensibility* (the perception that life events are structured, predictable, and explicable), *manageability* (the perception that adequate resources are available to meet life’s demands), and *meaningfulness* (the perception that life’s demands are challenges worthy of investment and engagement). Eriksson and Lindström’s (18) systematic review documented a consistent inverse association between sense of coherence and mental health symptoms across populations, and Schäfer et al.’s (19) meta-analysis confirmed sense of coherence as robustly protective against post-traumatic stress; construct validity has been supported in refugee samples (20). In a foundational study of 2,234 Southeast Asian refugees, sense of coherence mediated the effects of trauma exposure and resources on depression, anxiety, and psychosocial dysfunction (21). Although sense of coherence stabilizes in early adulthood, Antonovsky (16) held that it remains responsive to major changes in life patterns — a condition that forced migration unambiguously meets. Sense of coherence therefore offers a candidate mechanism through which both past trauma and ongoing post-migration stress translate into present mental health functioning. The present study examines the relative predictive strength of exposure to potentially traumatic events versus post-migration stress, and the mediation pathways through which sense of coherence influences the association between these predictors and mental health functioning. We focus on general mental health functioning — encompassing symptom distress, interpersonal relations, and social role performance — rather than disorder-specific symptomatology, because refugees and asylum seekers commonly carry mixed and subclinical symptom profiles (4) for which a broad functioning indicator is more informative.

We hypothesized that (a) post-migration stress and potentially traumatic events would each predict poorer functioning, with the former carrying substantial weight independent of trauma exposure, and (b) sense of coherence would mediate the associations of both stressors with functioning. Because PTE exposure represents past rather than ongoing adversity, its association with current functioning was expected to operate indirectly, through present-day sense of coherence and ongoing stressors; we therefore expected (c) the direct effect of PTE exposure to be substantially attenuated once sense of coherence and post-migration stress were taken into account.

## 2 Materials and methods

### 2.1 Study design and participants

Data for the present study were drawn from a project on which an earlier report has been published (22). Of 154 individuals enrolled, three withdrew consent (2.0%); the analytic sample comprised 151 refugees and asylum seekers living in the Netherlands (see **Table 1** for sample characteristics).

**Table 1:** Sample characteristics (N = 151).

Variable	n (%)	M (SD)
Sex		
Male	67 (44.4%)	
Female	84 (55.6%)	
Age, years		36.2 (10.8)
Length of stay, months		37.8 (15.9)
Region of origin^a^		
Middle East	107 (70.9%)	
Horn of Africa	17 (11.3%)	
Palestinian (Syria)	7 (4.6%)	
North Africa/Sub-Saharan Africa	13 (8.6%)	
Other	7 (4.6%)	
Mother tongue		
Arabic	99 (65.6%)	
Tigrinya	14 (9.3%)	
Farsi	11 (7.3%)	
Kurdish	11 (7.3%)	
Other	16 (10.6%)	
Residence permit		
Yes	127 (84.1%)	
No	24 (15.9%)	
Marital status		
Single	38 (25.2%)	
Married/cohabiting	100 (66.2%)	
Divorced/widowed	11 (7.3%)	
Other	2 (1.3%)	
Highest education level		
None or no diploma	8 (5.3%)	
Primary school	28 (18.5%)	
Secondary school	38 (25.2%)	
Vocational (MBO)	18 (11.9%)	
Higher professional (HBO)	20 (13.2%)	
University	38 (25.2%)	
Other	1 (0.7%)	

Length of stay range = 6–60 months (M = 3.2 years, SD = 1.3 years); age range = 18–63 years. Percentages may not sum to 100% due to rounding. ^a^Region of origin groupings: Middle East = Syria, Iran, Iraq, Yemen, Saudi Arabia; Horn of Africa = Eritrea, Ethiopia; Palestinian (Syria) = Palestinian Syrian respondents; North Africa/Sub-Saharan Africa = Egypt, Morocco, Sudan, Ghana, Nigeria, Sierra Leone, Rwanda, Uganda; Other = Turkey, Pakistan, Venezuela.

### 2.2 Measures

The instruments below were administered as part of a broader assessment battery; the present analysis focuses on potentially traumatic events, post-migration stress, sense of coherence, and mental health functioning.

#### 2.2.1 Life events checklist for DSM-5

Exposure to potentially traumatic events was assessed using the Life Events Checklist for DSM-5 (LEC-5) (23). For each of 16 event types, participants indicated whether the event had happened to them personally (yes/no). Endorsed events were summed into a cumulative trauma index of self-experienced potentially traumatic events (possible range 0–16), with higher scores indicating greater trauma load. Cross-cultural validation studies have demonstrated good test-retest reliability and convergent validity in European (24) and refugee populations (25, 26).

#### 2.2.2 Post-migration living problems

Post-migration living difficulties were assessed using the Dutch version of the Post-Migration Living Problems checklist (PMLP) (27, 28), a widely used index of post-migration stress. The instrument measures the extent to which a range of current life circumstances are experienced as problematic over the past 12 months. Respondents rated each item on a 4-point scale (1 = no, 2 = a little, 3 = much, 4 = very much)^1^. A total severity score was calculated as the sum of the 23 items (possible range 23–92), with higher scores indicating greater post-migration stress. The PMLP has been used internationally in refugee research, with initial studies among Tamil and Iraqi refugees providing evidence for construct validity and positive associations between PMLP domains and psychopathology (28–30). Internal consistency in the current sample was good (Cronbach’s α = .82).

#### 2.2.3 Sense of coherence scale-13

Sense of coherence was assessed using the 13-item short form of Antonovsky’s Sense of Coherence Scale (SOC-13) (16, 17). Items are rated on a 7-point semantic differential scale, with five items reverse-coded; the total sum score ranges from 13 to 91, with higher scores indicating a stronger sense of coherence. Although the scale captures three components — comprehensibility, manageability, and meaningfulness — Antonovsky (16, 17) intended a single global score and maintained that the components should not be examined separately. Contemporary psychometric evidence supports this, with very high inter-factor correlations and a dominant single-factor structure across diverse populations (32). The SOC-13 has demonstrated adequate validity in refugee populations (20). Internal consistency in the current sample was modest but acceptable (Cronbach’s α = .64), comparable to estimates reported in other refugee samples (e.g., α = .67–.74 in Eritrean refugees; 20).

#### 2.2.4 Outcome questionnaire-45.2

Mental health functioning was assessed using the Outcome Questionnaire-45.2 (OQ-45.2) (33, 34), a 45-item self-report measure capturing symptom distress (25 items), interpersonal relations (11 items), and social role performance (9 items). Items are rated on a 5-point scale (0 = never to 4 = almost always), with positively-worded items reverse-coded. The total score ranges from 0 to 180, with higher scores indicating greater distress and functional impairment; consistent with literature recommendations, only the total score was used in the present analyses. Total scores ≥ 63 are considered indicative of clinical-range functioning (33, 35). The OQ-45.2 has been validated in Dutch clinical and community samples (35). Internal consistency in the current sample was excellent (Cronbach’s α = .94).

### 2.3 Procedure

A cross-sectional survey was conducted in the Netherlands between January and December 2020 in collaboration with VluchtelingenWerk Nederland, Pharos, and ARQ Centrum’45 (22). The study received ethical approval from Utrecht University (reference: 19-087), with an addendum approved in April 2020 for COVID-19 modifications. Participants were recruited through convenience sampling. Inclusion criteria were: (1) age ≥ 18, (2) residing in the Netherlands six months to five years, and (3) sufficient proficiency in Dutch, English, or Arabic. Five research assistants (all fluent in Dutch and English; one a native Arabic speaker), trained in instrument administration and participant support, administered questionnaires in small groups (4–6) in private locations. During the spring 2020 lockdown, 23 participants were assessed individually via telephone or video call. Following informed consent, participants completed the battery in the presence of a research assistant, who clarified items when needed — supporting comprehension across languages and reducing the risk of misinterpretation associated with unsupervised self-report. Participants received a small financial compensation. Questionnaires not available in Arabic were translated and back-translated by a professional translation agency, following ITC (36) guidelines. Data were anonymized and stored securely.

### 2.4 Statistical analyses

We performed all analyses in IBM SPSS Statistics 31 and used Hayes’ PROCESS macro version 4.2 for the mediation analyses (37). All tests were two-tailed with α = .05.

We first ran descriptive frequency analyses to identify the most commonly endorsed PTEs and post-migration living problems. For participants with one missing item on a multi-item scale, we replaced the missing value with that participant’s mean across the remaining items: this concerned seven cases on the SOC-13 and three cases on individual PMLP items. After single-item imputation, all 151 participants had complete data on the study variables; no further case exclusion was required. We checked the assumptions for multiple regression and mediation: residuals were approximately normally distributed, all VIF values stayed below 1.2 (tolerance >.70), and no extreme outliers were detected.

To test hypothesis (a), we conducted a multiple regression with OQ-45.2 as the dependent variable and PTE exposure and post-migration stress entered together as predictors.

To test hypotheses (b) and (c), we ran three PROCESS Model 4 analyses: (1) PMLP → SOC → OQ-45.2; (2) PTE exposure → SOC → OQ-45.2; and (3) a parallel mediation model with PTE exposure as predictor and SOC and PMLP as simultaneous mediators of OQ-45.2. We tested indirect effects with percentile bootstrap 95% confidence intervals based on 5,000 resamples, and considered an indirect effect significant when the CI excluded zero. Because the data are cross-sectional, the mediation models test a hypothesized ordering rather than establishing temporal or causal precedence; the indirect effects reported below should be read as statistical decompositions of concurrent associations.

To identify potential confounders, we screened five demographic variables — sex, age, length of stay, educational level, and residence status (whether participants held a residence permit for the Netherlands at the time of assessment) — for their bivariate associations with the predictors, the mediator, and the outcome (**Table 2**). Only residence status was associated with the predictors as well as with the mediator and the outcome; we therefore entered residence status as a covariate in all three mediation models. The remaining variables were unrelated to the mediator and the outcome and were not retained, in order to preserve degrees of freedom in a sample of this size.

**Table 2:** Descriptive statistics and bivariate correlations for the four key study variables and demographic screening variables (N = 151).

Variable	M	SD	α	1	2	3	4
1. Trauma exposure (LEC-5)	3.32	2.74	—	—			
2. Post-migration stress (PMLP)	52.17	11.43	.82	.39***	—		
3. Sense of coherence (SOC-13)	56.22	10.68	.64	−.30***	−.43***	—	
4. Mental health functioning (OQ-45.2)	57.51	29.91	.94	.33***	.47***	−.57***	—
Demographic correlates							
Sex (female)				−.03	−.03	−.04	.01
Age				.12	.12	.16*	−.17*
Length of stay (months)				.06	.00	−.14	−.07
Residence status (no permit)				.28***	.37***	−.24**	.27***
Education – middle (vs. low)				−.07	.01	−.11	−.03
Education – high (vs. low)				.17*	.15	.04	−.12

N = 151. Trauma exposure was operationalized as the count of self-experienced events on the LEC-5 (range 0–12, Mdn = 3). Higher OQ-45.2 scores indicate poorer mental health functioning. Columns 1–4 correspond to the four numbered study variables; the upper block reports their intercorrelations, and the lower block reports the bivariate correlation of each demographic screening variable with variables 1–4. Residence status coded 1 = residence permit, 2 = no permit (higher = no permit); sex coded female = 1; educational level entered as two dummy variables (middle and high, each vs. low as reference; n = 150). Two-tailed significance tests. *p <.05, **p <.01, ***p <.001.

## 3 Results

### 3.1 Descriptive statistics

Sample characteristics are described in §2.1 and **Table 1**. Descriptive statistics for the four study variables are presented in **Table 2**. Participants reported a median of three self-experienced potentially traumatic events on the LEC-5 (observed range 0–12), with 84.1% having experienced at least one such event; item-level endorsement rates are presented in §3.2 and **Table 3**. On average, participants reported moderate post-migration stress (*M* = 52.17, *SD* = 11.43, possible range 23–92) and a moderate sense of coherence (*M* = 56.22, *SD* = 10.68, possible range 13–91). Mean OQ-45.2 scores (*M* = 57.51, *SD* = 29.91, possible range 0–180) approached the clinical cutoff of 63 used to differentiate clinical from non-clinical functioning (33, 35), indicating that the sample carried a substantial mental health burden.

**Table 3:** Most frequently endorsed potentially traumatic events (LEC-5).

PTE event	n ‘happened to me’	% ‘happened to me’	n responding
**Severe human suffering**	**61**	**41.2**	**148**
Combat or exposure to a war-zone	40	26.7	150
Assault with a weapon	35	23.2	151
Sudden, accidental death	30	20.0	150
Physical assault	28	18.7	150
Fire or explosion	27	17.9	151
Serious accident at work, home, or recreational	24	16.0	150
Life-threatening illness or injury	19	12.8	149
Transportation accident	19	12.7	150
Sexual assault	18	12.1	149
Captivity	14	9.4	149
Other unwanted sexual experience	12	8.1	149
Sudden violent death	12	8.1	149
Exposure to toxic substance	11	7.3	150
Natural disaster	10	6.8	147
**Serious injury/death you caused to someone else**	**5**	**3.3**	**150**

Events ordered by percentage. Endorsement reflects events reported as having happened to the participant personally. n Responding, number of participants who provided a valid response to that item. Bold, most and least frequently endorsed events.

### 3.2 Potentially traumatic events and post-migration living problems

#### 3.2.1 Potentially traumatic events

**Table 3** shows endorsement rates of self-experienced potentially traumatic events. Severe human suffering (41.2%), combat or exposure to a war-zone (26.7%), and assault with a weapon (23.2%) were the three most commonly endorsed events; respondent-caused serious injury or death (3.3%) was least commonly endorsed. Sexual assault was reported by 12.1% of participants and captivity by 9.4%.

In an exploratory comparison by residence status, participants without a residence permit reported a greater number of distinct potentially traumatic event types than those with a permit (M = 5.04, SD = 2.96 vs. M = 2.99, SD = 2.58; Mdn = 5 vs. 2; n = 24 vs. 127), consistent with the positive trauma–residence-status correlation (r = .28). With only 24 participants without a permit, the data were underpowered to compare individual event types, and no single event type accounted for the difference. Country of origin did not account for this pattern: trauma load did not differ across nationality groups in an exploratory one-way ANOVA (20 groups, most with few participants; F(19, 131) = 1.07, p = .39), and the largest groups (Syria, M = 3.48, n = 88; Iran, M = 3.67, n = 12; Eritrea, M = 2.33, n = 15) fell near the sample mean of 3.32.

#### 3.2.2 Post-migration living problems

Family-related concerns dominated the post-migration burden profile. The three most strongly endorsed PMLP problems — defined as items rated as *much* or *very much* of a problem (i.e., score ≥3 on the 4-point scale) — were *worries about family in country of origin* (78.8%, *M* = 3.33), *missing family* (73.5%, *M* = 3.17), and *uncertainty about the future* (70.9%, *M* = 2.95). Items related to legal-status uncertainty (e.g., *uncertainty about residence*, 29.8%; *fear of being returned*, 35.8%) were endorsed less strongly, consistent with the fact that 84.1% of participants held a residence permit at the time of assessment. **Supplementary Table 1** provides item-level statistics for all 23 items.

### 3.3 Patterns among study variables

Bivariate correlations (**Table 2**) revealed significant associations in expected directions. Both PTE exposure (*r* = .33, *p* <.001) and post-migration stress (*r* = .47, *p* <.001) were positively associated with poorer mental health functioning, indicating that higher trauma load and greater post-migration stress were each related to greater symptom burden on the OQ-45.2. Sense of coherence showed the strongest association with mental health functioning (*r* = -.57, *p* <.001), with higher sense of coherence corresponding to better functioning, and was negatively correlated with both PTE exposure (*r* = -.30, *p* <.001) and post-migration stress (*r* = -.43, *p* <.001). PTE exposure and post-migration stress were moderately positively correlated (*r* = .39, *p* <.001).

### 3.4 Predictors of mental health functioning

Both PTE exposure (β = .17, *p* = .026) and post-migration stress (β = .40, *p* <.001) significantly predicted poorer mental health functioning, with PMLP showing a standardized coefficient more than twice as large as PTE exposure. Together, the two predictors explained 24.3% of the variance in OQ-45.2 scores, F(2, 148) = 23.81, *p* <.001 (see **Table 4**).

**Table 4:** Multiple regression predicting mental health functioning.

Predictor	B	SE	β	t	p	95% CI
Constant	-3.14	10.09	—	-0.31	.756	[-23.07, 16.79]
PTE exposure	1.91	0.85	.17	2.24	.026	[0.23, 3.59]
Post-migration stress (PMLP)	1.04	0.20	.40	5.11	<.001	[0.64, 1.45]

N = 151. F(2, 148) = 23.81, p <.001; R² = .243; Adjusted R² = .233. VIF < 1.2 for all predictors. PMLP entered as sum score (range 23–92).

### 3.5 Sense of coherence as a mediating pathway

#### 3.5.1 Post-migration stress and functioning via sense of coherence

PMLP was significantly negatively associated with sense of coherence (*b* = -0.37, *SE* = 0.07, *p* <.001). Sense of coherence significantly predicted functioning (*b* = -1.26, *SE* = 0.20, *p* <.001). The indirect effect through sense of coherence was significant [*b* = 0.47, 95% CI (0.23, 0.79)], accounting for 42.2% of the total effect. The direct effect remained significant (*b* = 0.64, *p* = .001), indicating partial mediation.

#### 3.5.2 Trauma exposure and functioning via sense of coherence

PTE exposure was significantly negatively associated with sense of coherence (*b* = -0.99, *SE* = 0.31, *p* = .002). The indirect effect through sense of coherence was significant [*b* = 1.40, 95% CI (0.29, 2.43)], accounting for 46.3% of the total effect. The direct effect remained significant (*b* = 1.62, *p* = .037), indicating partial mediation.

#### 3.5.3 Joint pathways through sense of coherence and post-migration stress

**Figure 1:**
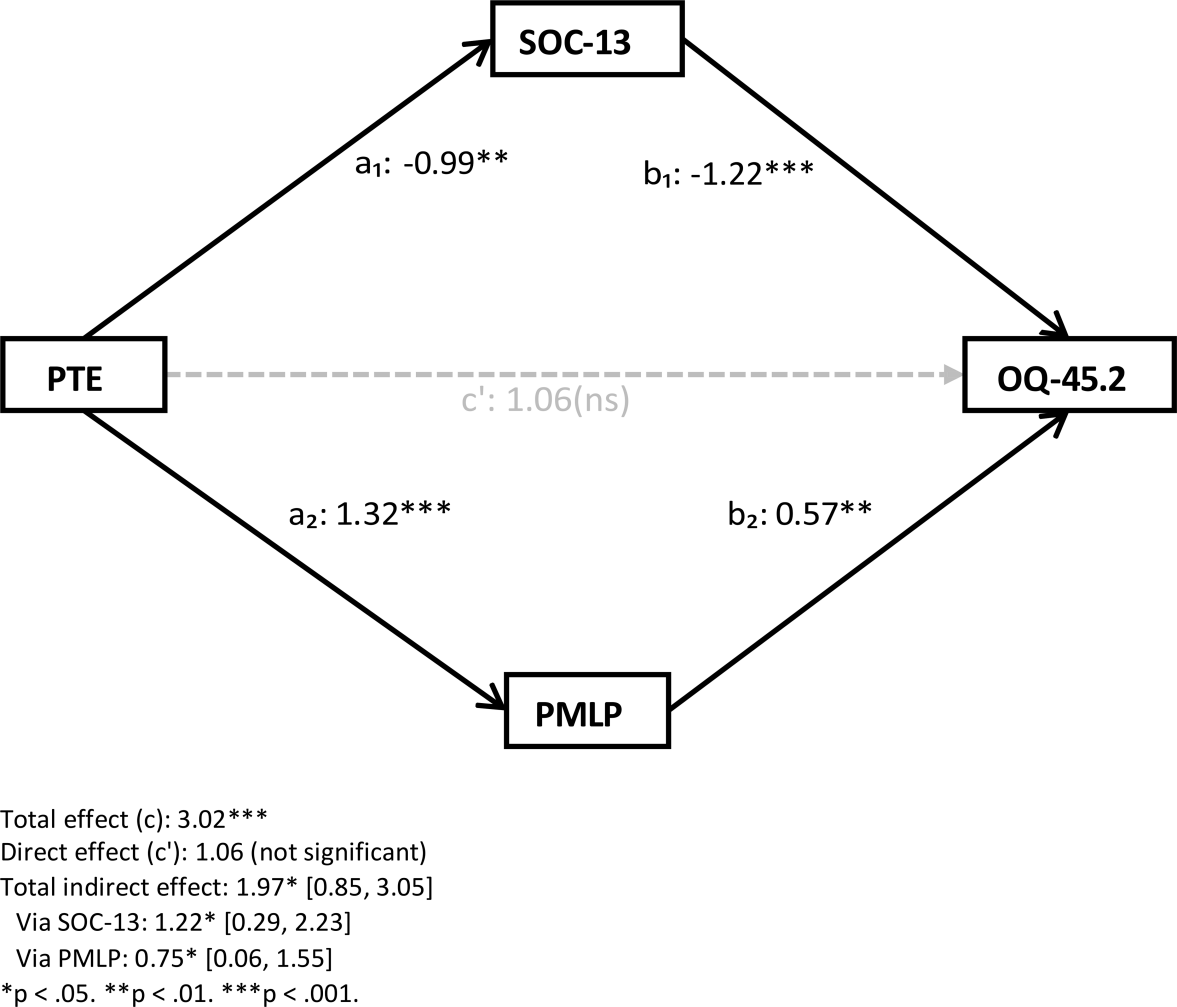
Parallel mediation model: PTE exposure on mental health functioning via SOC-13 and PMLP (N = 151). N = 151. PROCESS Model 4 (Hayes), 5,000 bootstrap samples. Solid arrows, significant (*p <.05, **p <.01, ***p <.001). Dashed arrow, non-significant direct effect. Coefficients are unstandardized and adjusted for residence status.

The parallel mediation analysis examined whether the PTE–functioning association operates entirely through sense of coherence and PMLP as parallel mediators (see **Table 5**; **Figure 1**).

**Table 5:** Parallel mediation model: PTE effects on functioning via SOC and PMLP.

Pathway	B	SE	t	p	95% CI
Paths to mediators					
PTE → SOC-13 (a_1_)	-0.99	0.31	-3.17	.002	[-1.61, -0.38]
PTE → PMLP (a_2_)	1.32	0.31	4.22	<.001	[0.70, 1.94]
Paths to mental health functioning					
SOC-13 → OQ-45.2 (b_1_)	-1.22	0.20	-6.06	<.001	[-1.62, -0.82]
PMLP → OQ-45.2 (b_2_)	0.57	0.20	2.82	.006	[0.17, 0.97]
PTE → OQ-45.2 (direct, c′)	1.06	0.78	1.36	.177	[-0.48, 2.60]

N = 151. Hayes’ PROCESS (Model 4), 5,000 bootstrap samples, two-tailed. Coefficients are adjusted for residence status.

The total effect of PTE exposure on mental health functioning was significant [c = 3.02, SE = 0.86, p <.001, 95% CI (1.32, 4.73)]. When both mediators were included, the direct effect of PTE exposure became non-significant (*b* = 1.06, *p* = .177), while the total indirect effect remained significant [*b* = 1.97, 95% CI (0.85, 3.05)]. The indirect effect via sense of coherence was significant [b = 1.22, 95% CI (0.29, 2.23), 40.2% of total effect], as was the indirect effect via PMLP [b = 0.75, 95% CI (0.06, 1.55), 24.9% of total effect]; the two specific indirect effects were comparable in magnitude. Together, these specific indirect effects accounted for 65.1% of the total effect. The direct effect was non-significant while the total indirect effect was significant, indicating that the PTE–functioning association was mediated by sense of coherence and PMLP, with the direct effect no longer statistically significant in this cross-sectional sample.

## 4 Discussion

### 4.1 Summary of principal findings

This study examined how trauma exposure, post-migration stress, and sense of coherence together relate to mental health functioning among refugees and asylum seekers in the Netherlands. Three findings stand out. First, sense of coherence statistically mediated the associations of both trauma exposure and post-migration stress with poorer functioning. Of the three study variables, it was the one most strongly associated with functioning, both as a correlate (r = –.57) and as a mediating pathway. Second, when sense of coherence and post-migration stress were entered as parallel mediators, trauma exposure was no longer directly associated with functioning. Third, post-migration stress was substantially more strongly associated with functioning than trauma exposure (β = .40 vs. .17; together they explained 24% of the variance), underscoring the ongoing burden of displacement-related adversity. Read together, these findings locate refugee mental health within an ecological rather than narrowly trauma-centric frame: present functioning reflects not past exposure in isolation but its interplay with the ongoing post-migration context and the capacity to keep that context comprehensible, manageable, and meaningful.

### 4.2 The mediating role of sense of coherence

Both past trauma and ongoing post-migration stress were associated with mental health functioning partly through sense of coherence — through whether the world remains comprehensible, manageable, and meaningful (16). Post-migration stress was, in addition, associated with functioning directly, beyond this indirect pathway.

This is consistent with the broader salutogenic literature (31): Eriksson and Lindström’s (18) systematic review found a strong and consistent inverse association between sense of coherence and mental health symptoms across populations, and Schäfer et al.’s (19) meta-analysis showed sense of coherence to be robustly protective against post-traumatic stress. What our findings add is that, in our sample, sense of coherence was the construct through which both past trauma and ongoing post-migration stress were most strongly statistically mediated, indicating a shared indirect association rather than a demonstrated causal pathway.

### 4.3 The weight of present context

Post-migration stress showed a substantially stronger association with mental health functioning than past trauma exposure, in line with prior meta-analytic and review evidence (9, 12, 13). This asymmetry — that ongoing post-migration stressors weigh more heavily on current functioning than discrete past traumatic events — is precisely what Conservation of Resources theory (15) predicts. Post-migration stressors are continuous and cumulative: uncertain status, restricted opportunities, and prolonged separation from family persist day after day, drawing down the very resources that recovery from prior adversity would require (14). In our data, this burden was predominantly relational rather than legal or material: worry about and separation from family in the country of origin were the most strongly endorsed difficulties, consistent with prior work in resettled samples (10, 11). We postulate that the continuing weight of these burdens, often invisible to the host society, deserves wider recognition.

Sense of coherence does not develop in isolation: although it is largely an internally driven disposition, it is shaped by consistent life experiences and opportunities to contribute to the community. This is illustrated by work on social capital, defined as the resources people derive from their relationships, networks, and the trust and reciprocity within them (45, 46), which has been shown to buffer against negative mental health outcomes following adversity (39). Consistent with this, in an earlier analysis of the present sample, van Sint Fiet and colleagues (22) found that cognitive social capital was associated with mental well-being entirely through sense of coherence. The precise relationship between social capital and sense of coherence falls outside the scope of the present paper, but warrants attention in future research on the sense-of-coherence pathway identified here.

### 4.4 Societal and policy implications

Because sense of coherence is shaped by external conditions — ongoing insecurity, for instance, can erode it over time (40) — supporting it is not solely an individual matter. It may therefore be beneficial to invest in societal initiatives and policies that address its components, for example by stimulating meaningfulness through positioning refugees as contributors to these initiatives rather than only as recipients. Consistent with this, loneliness and a lack of social contact were each endorsed by more than half of participants; their prominence marks social connectedness as a domain warranting attention in post-migration support (41, 43).

The relative weight of post-migration stress in these analyses does not diminish the importance of trauma-focused care: refugees presenting with persistent post-traumatic stress symptoms continue to warrant prompt access to evidence-based treatment. The present findings, drawn from a cross-sectional analysis, do not license specific intervention recommendations for sustaining sense of coherence in this population. Because the functional impact of past trauma here operated through present mechanisms rather than independently, what the findings support is sustained attention to the sense-of-coherence pathway identified in this sample. That pathway is a meaningful focus for the societal and policy responses considered here, and a priority for the longitudinal and intervention research needed to test them.

### 4.5 Strengths and limitations

The present study has several strengths. It addressed past trauma and ongoing post-migration stress in the same model, allowing their relative contributions to functioning to be compared rather than estimated in separate samples. The instruments used were well-validated for their respective constructs, and the analytic approach combined multiple regression with parallel mediation to test both the additive and the mechanistic structure of the associations. Questionnaires were administered in person, with research-assistant support to clarify items when needed, mitigating comprehension-related concerns common to unsupervised self-report assessment in multilingual samples. The sample, while modest in size, was specific to refugees and asylum seekers in the Netherlands and provided a coherent context for testing the mediating role of sense of coherence.

A further feature of the design deserves consideration as both a strength and a limitation. The OQ-45.2 captures general mental health functioning rather than PTSD-specific symptomatology, and no PTSD-specific instrument was administered. On the one hand, this is consistent with the recognition that trauma-related psychopathology extends beyond PTSD diagnostic criteria, particularly in populations exposed to cumulative and relational threats (42), and it positions the outcome at the level of broad functional impairment rather than a single diagnostic category. In capturing not only symptom distress but also interpersonal and social-role functioning, the measure indexes everyday adaptation with greater ecological validity than disorder-specific symptom counts would allow. On the other hand, it constrains the interpretation of the findings. The question whether the pattern found in this study upholds trauma-specific outcomes remains a separate empirical question. The asymmetry may be sample-dependent: in subgroups with severe trauma profiles, or earlier in the post-migration trajectory, past trauma may weigh more heavily relative to post-migration stress.

Several limitations should be considered. First, the cross-sectional design precludes causal inference, leaving the assumed temporal ordering of the mediation models unconfirmed, and cannot capture dynamic processes such as trauma reactivation, in which post-migration experiences may reactivate pre-migration trauma responses. Second, the sample was recruited through convenience sampling. Third, the LEC-5 captures lifetime exposure as a count of self-experienced events but does not capture severity or chronicity. Fourth, all constructs were assessed by self-report at a single time point, so common method variance cannot be ruled out as a contributor to the observed associations. The instruments differ in format and response scale, however, making a single response style an unlikely explanation for the pattern of findings. Nonetheless, single-time-point self-report remains a limitation, and replication with multi-method or multi-informant designs is recommended. Fifth, internal consistency of the SOC-13 in the present sample was modest (α = .64), although comparable to estimates reported in other refugee samples (20). Because measurement error attenuates observed associations, the modest reliability is likely to have biased the sense-of-coherence effects toward zero; the significant indirect effects via sense of coherence should therefore be read as conservative estimates rather than as artifacts of low reliability.

A final consideration concerns the decision to adjust the mediation models for residence status. This covariate was retained because it was associated with both stressors, with sense of coherence, and with mental health functioning, and because participants without a residence permit differed from those with a permit on post-migration stress, sense of coherence, functioning, and length of stay, though not on age, sex, or education. Adjusting for residence status therefore guards against confounding by legal insecurity. Legal insecurity is, however, itself a component of the post-migration burden rather than a factor wholly external to it; residence status was moderately associated with post-migration stress (r = .37). Controlling for it may consequently partial out variance that belongs to the post-migration-stress construct, so the adjusted estimate of the post-migration-stress pathway is, if anything, conservative. The indirect effects that remained significant in the adjusted models should be read in that light.

One descriptive finding warrants brief comment. Respondents without a residence permit reported more types of potentially traumatic events than those with a permit. This is difficult to interpret, as trauma exposure is largely pre-migration and such differences would not be expected. The pattern was not explained by country of origin, which was related to residence status but not to trauma load in this sample (§3.2). It may reflect differences in migration trajectories, asylum procedures, or other unmeasured factors. Further research is needed to examine this.

Future research could extend this work in four directions. Longitudinal designs would permit tests of whether changes in post-migration conditions produce corresponding changes in sense of coherence and functioning over time. Studies combining the OQ-45.2 with disorder-specific instruments would allow the relative weight of past trauma versus ongoing stress to be examined across multiple outcome domains within the same sample. Intervention research could examine whether sense of coherence is a modifiable mechanism — and whether interventions targeting it produce corresponding gains in mental health functioning. Future work could also examine the association between religiosity or spirituality, not assessed in the present study, andsense of coherence in this population, given evidence that this association may be particularly strong in Muslim-majority and Middle Eastern contexts (47, 48).

## 5 Conclusion

Among refugees and asylum seekers in the Netherlands, sense of coherence emerged as the strongest indirect, statistically mediating link between both past trauma and ongoing post-migration stress and mental health functioning. Post-migration stress weighed more heavily than past trauma exposure in predicting mental health functioning, with family-oriented and existential stressors dominating the burden profile. These findings highlight sense of coherence — a personal-resilience construct that cannot be entirely separated from external conditions — as a meaningful focus for understanding mental health in this population. Longitudinal research is needed to confirm these pathways and to clarify whether — and if so, how — sense of coherence develops and changes over the post-migration period.

## Data Availability

The raw data supporting the conclusions of this article will be made available by the authors, without undue reservation.

## Footnotes

1 The Dutch adaptation (28) uses a 1–4 response format, omitting the lowest anchor of the original 0–4 PMLP scale (11, 27) while preserving four ordinal severity levels.

## Supplementary material

Supplementary Table 1 Item-level descriptive statistics for the post-migration living problems checklist (N = 151), ranked by mean severity.
