# Supplementary material for: Sense of coherence as a pathway linking war trauma and post-migration stress to mental health functioning among refugees and asylum seekers in the Netherlands
Source: Front Psychiatry. 2026 Jul 15;17:1890660. doi: 10.3389/fpsyt.2026.1890660 (PMC13418565; doi:10.3389/fpsyt.2026.1890660)
Supplement: Supplementary Table 1 — Item-level descriptive statistics for the post-migration living problems checklist (N = 151), ranked by mean severity. [file Table1.docx]

# Supplementary Table 1

**Table S1.** *Item-level descriptive statistics for the Post-Migration Living Problems checklist (N = 151), ranked by mean severity.*

| **Rank** | **Item** | **M** | **SD** | **% rated ≥3** |
| --- | --- | --- | --- | --- |
| 1 | Worries about family in country of origin | 3.33 | 1.08 | 78.8 |
| 2 | Missing family | 3.17 | 1.20 | 73.5 |
| 3 | Uncertainty about the future | 2.95 | 1.05 | 70.9 |
| 4 | Inability to return home in case of emergency | 2.87 | 1.31 | 63.6 |
| 5 | Language difficulties | 2.72 | 1.06 | 59.6 |
| 6 | Loneliness | 2.62 | 1.22 | 53.6 |
| 7 | Unemployment | 2.56 | 1.24 | 55.6 |
| 8 | Lack of social contacts | 2.53 | 1.04 | 55.0 |
| 9 | Financial problems (self/own family) | 2.49 | 1.04 | 52.3 |
| 10 | Financial problems (obligations to family) | 2.44 | 1.14 | 47.0 |
| 11 | Working below educational level | 2.36 | 1.25 | 47.7 |
| 12 | Health problems | 2.16 | 1.17 | 39.1 |
| 13 | Worries about political friends in country of origin | 2.15 | 1.31 | 38.4 |
| 14 | Fear of being returned | 2.07 | 1.19 | 35.8 |
| 15 | Housing problems | 1.99 | 1.22 | 34.4 |
| 16 | Uncertainty about residence | 1.97 | 1.19 | 29.8 |
| 17 | Discrimination (general) | 1.79 | 0.93 | 23.2 |
| 18 | Lack of religious meetings | 1.77 | 1.10 | 25.8 |
| 19 | Discrimination (in actions) | 1.76 | 0.88 | 20.5 |
| 20 | Lack of contact with people of same religion | 1.75 | 1.04 | 24.5 |
| 21 | Lack of privacy | 1.71 | 1.02 | 21.2 |
| 22 | Discrimination (in words) | 1.63 | 0.86 | 14.6 |
| 23 | Lack of safe environment for children | 1.38 | 0.83 | 11.9 |

*Note.* Items rated on a 4-point scale (1 = no, 2 = a little, 3 = much, 4 = very much). Following standard PMLP scoring conventions (27, 28), items rated ≥3 were classified as endorsed problems. All 23 items verified against SPSS output (N = 151 valid responses for all items).
